# Post-Thyroidectomy Hematoma: Risk Factors To Be Considered for Ambulatory Thyroidectomy

**DOI:** 10.7759/cureus.31539

**Published:** 2022-11-15

**Authors:** Saad M Alqahtani, Hanan R Al-sohabi, Areej A Alfattani, Yousef Alalawi

**Affiliations:** 1 Department of Surgery, College of Medicine, Majmaah University, Al-Majmaah, SAU; 2 Department of Surgery, King Salman Armed Forces Hospital in North-Western Region, Tabuk, SAU; 3 Department of Biostatistics, Epidemiology, and Scientific Computing, King Faisal Specialist Hospital and Research Centre, Riyadh, SAU; 4 Department of Surgery, King Salman Armed Forces Hospital In the North-Western Region, Tabuk, SAU

**Keywords:** risk factor, outpatient thyroidectomy, hematoma, complications of thyroidectomy, ambulatory thyroidectomy

## Abstract

Introduction: Post-thyroidectomy hematoma is a serious, potentially life-threatening complication and it is the most frequent indication for reoperation. In this study, we aimed to identify the risk factors and prevalence of post-thyroidectomy hematoma and perform a literature review of the incidence of post-thyroidectomy hematoma at other centers in Saudi Arabia.

Methods: We retrospectively enrolled 372 consecutive patients who had undergone thyroidectomy between January 2015 and December 2020. Patients with bleeding disorders were excluded from the study. Data were analyzed using SPSS software, version 20.0 (IBM Corp., Armonk, NY).

Results: Three female (0.8%) patients developed a hematoma after thyroidectomy. Two patients developed a hematoma 24 hours after surgery and were treated conservatively. The third patient developed a hematoma within two hours of surgery and required surgical intervention. None of the patients required a tracheostomy, and there was no mortality. No significant association was found between age, sex, final pathology, the extent of thyroidectomy, and risk of hematoma.

Conclusion: A post-thyroidectomy hematoma is a rare but dangerous complication. Identifying the risk factors for hematoma formation is of great importance, particularly when considering outpatient thyroidectomy. A large prospective multicenter study is needed for further investigation.

## Introduction

The thyroid gland is a highly vascularized organ, and thyroidectomy is a common and relatively safe procedure. However, like other procedures, there is a risk of postoperative complications [1,2]. Hypoparathyroidism, recurrent laryngeal nerve paralysis, loss of high-pitched voice, hematoma, and seroma are well-known post-thyroidectomy complications. Among these, hypocalcemia is considered the most common cause of hospital readmission, and hematoma is the most frequent indication for reoperation [3].

Although uncommon, a post-thyroidectomy hematoma is a serious, potentially life-threatening complication [4] with an incidence of 0.7 to 4.7% [1,5], and can reportedly reach 6.5% [2]. This is extremely important when considering ambulatory thyroidectomy [6]. Post-thyroidectomy hematoma formation may be related to patient predisposition, surgical technique, and thyroid pathology [6].

Post-thyroidectomy hematoma can present as neck swelling, choking sensation, neck pressure, respiratory distress, wound drainage, dysphagia, or difficulty with phonation [7]. In severe cases, cerebral anoxia with major neurological complications and even death may occur [1,5].

Post-thyroidectomy hematoma commonly occurs within the first six hours after surgery; however, it can occur even up to 24 hours after surgery [6]. The risk factors for post-thyroidectomy hematoma include coexisting morbidities (diabetes mellitus {DM}, obesity, chronic kidney disease, chronic obstructive pulmonary disease, congestive heart failure, bleeding disorder, sleep apnea, and hypertension), male sex, older age, certain thyroid diseases (Graves’ disease, malignancy, and Hashimoto's thyroiditis), retrosternal goiter, the extent of thyroidectomy, resected thyroid volume, redo-surgeries, drain use, high postoperative blood pressure, smoking, medications (antithrombotic agent), and prior neck radiation [1,5,8].

This study aimed to identify the risk factors and prevalence of post-thyroidectomy hematoma in our setting. Such factors should be considered when selecting patients for ambulatory thyroidectomy and those at higher risk for hematoma formation. Additionally, we have performed a literature review of the incidence of post-thyroidectomy hematoma at other centers in Saudi Arabia.

## Materials and methods

This retrospective study was approved by the ethics committee at King Salman Armed Forces Hospital (KSAFH) in North-Western Region, Tabuk, Saudi Arabia. (No: KSAFH-REC-2017166). Informed consent from patients was not required, as secondary data were used with no direct patient contact.

The study included all patients who underwent thyroidectomies between January 2015 and December 2020 at KSAFH, the largest referral hospital in the Tabuk region. Patients with bleeding disorders were excluded from the study. Data collected included demographic information, final histopathologic type, and extent of thyroidectomy. The latter refers to the following: (1) Hemithyroidectomy, which involves the removal of one thyroid lobe and the isthmus; (2) Completion thyroidectomy, which is the removal of the remaining part of the thyroid gland; (3) Subtotal thyroidectomy, which involves the removal of one lobe of the gland, isthmus, and the majority of the other lobe; and (4) Total thyroidectomy, which is the removal of the entire thyroid gland. A drain was routinely placed in most total thyroidectomy and neck dissection cases.

Statistical analysis

Data were analyzed using the Statistical Package for the Social Sciences (SPSS) software for Windows, version 20.0 (IBM Corp., Armonk, NY). The Mann-Whitney U test was used to compare the age between the hematoma groups, which was presented as the median and interquartile range (IQR). Frequencies and proportions were used to describe nominal data. Fisher’s exact test was used to compare the nominal variables between the hematoma groups because of the low cell count in the contingency tables. Differences were considered statistically significant at p < 0.05.

## Results

In total, 372 thyroidectomies were performed during the study period. There were 311 (83.63%) female and 61 (16.4%) male patients. Their ages ranged from 13 to 95 years old. The final pathology was benign in 205 (55.11%) patients and malignant in 167 (44.89%). There were 222 total thyroidectomies (59.68%), 70 right hemithyroidectomies (18.81%), 52 left hemithyroidectomies (13.98%), 21 completion thyroidectomies (5.65%), and seven subtotal thyroidectomies (1.88%).

Three female patients developed hematomas (0.8%). Two patients developed hematomas after 24 hours of surgery and were treated conservatively. The third patient developed a significant hematoma within two hours of surgery, which necessitated surgical intervention. There was no mortality, and no patient required a tracheostomy.

Table 1 shows that there were no significant associations between age, sex, final pathology, surgery type, and hematoma risk. The characteristics of the patients with hematoma, various risk factors for hematoma formation post-thyroidectomy, hospital stay, and management are summarized in Table 2.

**Table 1:** Associations of patient demographics, final pathology, and extent of thyroidectomy with the risk of hematoma formation *Significant at p <0.05; IQR: interquartile range

		Hematoma			
		No N=369	Yes N=3	P-value^*^	
Age	Median (IQR)	39(15)	38(21)	0.94	
Gender	Female	308 (83.47%)	3 (100%)	1.00	
	Male	61 (16.53%)	0 (0.00%)		
Final Pathology	Benign	203 (55.01%)	2 (66.67%)	1.00	
	Malignant	166 (44.99%)	1 (33.33%)		
Extent Of thyroidectomy	Total thyroidectomy	221 (59.89%)	1 (33.33%)	0.260	
	Right hemithyroidectomy	69 (18.70%)	1 (33.33%)		
	Left hemithyroidectomy	52 (14.09%)	0 (0.00%)		
	Sub-total thyroidectomy	7 (1.90%)	0 (0.00%)		
	Completion thyroidectomy	20 (5.42%)	1 (33.33%)		

**Table 2:** Features of patients with post-thyroidectomy hematoma MNG: multinodular goiter, PTC: papillary thyroid carcinoma

Variables	Patient 1	Patient 2	Patient 3
A- When was the hematoma developed?	Next day (Mild-moderate hematoma)	Next day (Mild-moderate hematoma)	within 2 hours post operatively
B- Risk Factors			
Age	31	38	52
Gender	Female	Female	Female
Thyroid function status	Hypothyroidism	Euthyroid	Euthyroid
Diabetes mellites	No	No	No
Hypertension	No	No	No
Chronic kidney disease	No	No	No
Obesity (BMI)	23.3	35	28.2
Large goiter (or retrosternal)	No	Large goiter	Large goiter
Type of surgery	Completion thyroidectomy	Right Hemi-thyroidectomy	Total thyroidectomy
Presence of adhesions during surgery	Yes	No	Yes
Type of pathology	PTC and MNG	MNG	MNG
Neck dissection	Left neck dissection	No	No
Graves' Disease	No	No	No
Anticoagulants	No	No	Yes
Bleeding disorder	No	No	No
Previous neck radiation	No	No	No
Prior thyroid surgery	Yes, subtotal thyroidectomy	No	No
Use of energy-based vessel sealing devices (harmonic scalpel or LigaSure or bipolar)	Yes	Yes	Yes
Use of the hemostatic agent Surgicel	Yes	Yes	Yes
Use of a drain	No	Yes	Yes
C-Management of Hematoma			
Mortality	No	No	No
Tracheostomy	No	No	No
Surgical intervention of hematoma	No	No	Exploration, evacuation of hematoma and control the bleeding
Blood transfusion	No	No	No
Source of the post thyroidectomy bleeding	Treated conservatively	Treated conservatively	Middle thyroid vein
D-Hospital Stay	3 days	3 days	4 days
E- Blood pressure pre-, intra- and post-operatively:	Pre: 91/60	Pre:111/52	Pre :104/56
	Intra :120/90	Intra :110/60	Intra :120/70
	Post: 114/70	Post: 100/60	Post :108/89

## Discussion

The risk of hematoma formation in our cohort was 0.8%, which is in line with previous studies [1,4,5,8]. Table 3 shows the incidence of post-thyroidectomy hematomas in different cities in Saudi Arabia [9-13].

**Table 3:** Prevalence of post-thyroidectomy hematoma among the different cities in Saudi Arabia. * NR; not reported

Authors	Reference	Year	City	Number of total patients	Patients with Hematoma	Treatment	Mortality
Bawa et al.	[9]	2021	Bisha	339	3(1.4%)	Two were treated surgically & one conservatively	None
Alshareef et al.	[10]	2020	Makkah	140	1(5%)	NR	NR
Al-Hakami et al.	[11]	2019	Jeddah	456	3(0.65)	NR	NR
Alharbi and Ahmed	[12]	2018	Jazan	320	4(1.25%)	Three were treated surgically	1 died due to large wound hematoma
Alshahrani et al.	[13]	2013	Riyadh	88	1(1.1)	Conservatively	None
Present study		2022	Tabuk	372	3 (0.8)	One was treated surgically & two conservatively	None

Although hematoma is frequently seen within the first six hours after surgery, bleeding can still occur up to 24 hours after surgery [6]. Two of our patients developed hematomas 24 hours post-surgery, and the third patient developed a significant hematoma within two hours of surgery. Notably, in 55-90 % of cases, the source of bleeding has been identified [7]. The most common locations for significant postoperative bleeding are the superior thyroid artery, inferior thyroid artery, thyroid ima, arteries at the anterolateral junction of the trachea and cricoid cartilage, tiny arteries in the ligament of Berry, and medial cricothyroid vessels [7]. In our study, the source of bleeding in patient 3 was the middle thyroid vein.

Comorbidities (such as DM and hypertension), male sex, older age, Graves’ disease, thyroid cancer, Hashimoto's thyroiditis, retrosternal goiter, thyroidectomy extent, redo-surgeries, drain use, smoking, antithrombotic agents, and prior neck radiation are well-known risk factors for post-thyroidectomy hematoma formation [1,5,8]. Table 2 shows the various risk factors for post-thyroidectomy hematoma formation in our study.

Despite its rarity, a post-thyroidectomy hematoma is a potentially fatal complication. In recent years, the number of outpatient thyroidectomy procedures has increased, as they are cost-effective and reduce hospitalization. Furthermore, the mortality rate in patients with post-thyroidectomy hematoma is three times higher than that in patients without hematoma [6]. Therefore, the risk factors for post-thyroidectomy hematoma formation should be identified [4,6].

Chereau et al. proposed a neck hematoma scoring system for ambulatory thyroidectomy. Patients with a score of ≤ 1 were considered candidates for ambulatory surgery. Furthermore, they found that male patients taking vitamin K antagonists and/or patients with DM were not candidates for ambulatory thyroidectomy [5].

Some preventive measures that could lower the risk of post-thyroidectomy hematomas can be taken before, during, or after surgery. Preoperatively, hypertension and hyperthyroidism should be controlled. Intraoperatively, good hemostasis, performing the Valsalva maneuver, using an ultrasonic or radiofrequency scalpel, and loosening the strap muscle re-approximation can reduce the risk of hematoma formation. Keeping the strap muscle re-approximation loose allows for the earlier detection of hematomas in the subcutaneous tissue. Controlling postoperative high blood pressure, nausea, and vomiting as well as using deep anesthesia extubation to reduce coughing are recommended [2,5,7].

It is uncertain whether drain use increases the risk of hematoma formation. According to Smith and Coughlin, suction drainage increases the risk of bleeding [14]; however, another study found that drain use has no effect on the postoperative risk of hematoma and seroma formation [2]. In our study, a drain had been placed in two patients. The use of energy vessel sealant devices reportedly decreases the risk of post-thyroidectomy hematomas [8]. Notably, sealing devices were used in the three patients who had developed a hematoma.

If acute respiratory distress occurs, the wound should be opened immediately at the bedside. However, this is usually only required in 0-25% of cases. Surgical exploration in the operating room in the absence of respiratory distress is recommended [7]. In the absence of compressive symptoms with delayed hematoma formation, conservative management is recommended and hematoma usually resolves within 3-6 weeks [7]. Two of our patients had no compression symptoms, had stable vitals, and were treated conservatively. In contrast, the third patient developed a large hematoma within two hours of surgery and experienced respiratory distress, necessitating immediate surgical intervention (evacuation and control of the bleeding source).

Our study had several strengths. First, we compared the incidence of post-thyroidectomy hematomas among different centers in Saudi Arabia. Second, it sheds light on the different risk factors for hematoma formation in our patients and in previous studies; thus, they will be considered in the future for our institution's planned “Outpatient Thyroidectomy” program. On the other hand, it had limitations such as a retrospective nature and a single-center study. As a result, a large prospective study is required.

## Conclusions

Despite the fact that we observed a low incidence of hematoma, the current study sheds light on a potentially fatal post-thyroidectomy complication. It also provides a review of the literature on various potential risk factors that should be considered when selecting patients for ambulatory thyroidectomy and those at higher risk for hematoma formation.
